# Can Nutritional Adequacy Help Evade Neurodegeneration in Older Age? A Review

**DOI:** 10.7759/cureus.10921

**Published:** 2020-10-12

**Authors:** Uvie Ajibawo-Aganbi, Sania Saleem, Seyad Zulficar Ali Khan, Swathi Veliginti, Maria V Perez Bastidas, Rayan M Lungba, Ivan Cancarevic

**Affiliations:** 1 Health Sciences, California Institute of Behavioral Neurosciences & Psychology, Fairfield, USA; 2 Internal Medicine, California Institute of Behavioral Neurosciences & Psychology, Fairfield, USA; 3 Family Medicine, Ministry of Health Oman, Salalah, OMN; 4 Pulmonary Research Department, California Institute of Behavioral Neurosciences & Psychology, Fairfield, USA

**Keywords:** nutrition, brain degeneration, neurodegeneration

## Abstract

There is an increase in susceptibility to chronic and debilitating diseases with aging. The reason for the underlying neuronal degeneration and normal aging of the brain remains elusive. Different research studies have been conducted to discover how the brain degenerates and the importance of vitamins' role in the neurocognitive decline. Comprehensive literature research was conducted using all relevant data available from PubMed and Google scholar for this article. There has been evidence linking the consumption of essential nutrients to preventing the disease conditions that result in cognitive decline. This article provides the latest scientific advances specific to how dietary nutrients and non-nutrient may affect cognitive aging. An adequate supply of nutrients like vitamin B2 (riboflavin), vitamin B12, vitamin E, essential fatty acid (omega-3 fatty acid), and flavonoids play a vital role in ensuring healthy aging, enhancing memory, and strengthening neuroprotection. These nutrients help in neurodegenerative diseases like Alzheimer's disease and Parkinson's. We recommend more research studies to determine the underlying mechanism of how these essential nutrients work in the prevention of cognitive decline. These studies will help provide the evidence needed for new dietary recommendations for combating these diseases that often affect aging patients.

## Introduction and background

The brain, which is the powerhouse for our existence and functioning, is the most complex organ in a vertebrate's body. In the human cerebellum, the estimated number of neurons is 55-70 billion, and the cerebral cortex contains about 14-16 billion neurons [1,2]. Different factors, including nutrition, influence brain development. Various sources suggest a connection between improved nutrition and optimal brain function. Nutrients provide building blocks that play a significant role in cell proliferation, deoxyribonucleic acid synthesis, neurotransmitter, and hormone metabolism, and are essential constituents of enzyme systems in the brain [3]. The brain is an organ resistant to structural changes induced by exogenous factors. Research studies have shown that the brain responds to diet changes by altering neurotransmitter synthesis, thereby affecting the neuroendocrine system over various physiological events [4]. For many years it was not entirely accepted that food played a role in the brain structure and function. 

A child's full genetic potential for physical growth and mental development may be compromised due to dietary deficiencies [5]. The brain development is resistant to permanent damage from protein-energy malnutrition (PEM) [6]. However, specific nutrients play crucial roles in the function of the brain. Iodine deficiency, the most widespread nutrient deficiency, causes endemic cretinism, associated with deaf-mutism and cerebral palsy. Iodine deficiency during pregnancy results in an irreversible impairment of brain development at a critical stage [6]. About three-quarters of the total world population lives in the tropics but consumes only 6% of worldwide food production and contributes 15% of net revenue, leading to inadequate daily caloric intake compared to the rest of the world. One-third of the world's population consumes insufficient iodine, which increases the risk for mental retardation and deafness because of maternal hypothyroidism [7].

One of the most significant risk factors of neurodegenerative disease is old age. Research has been conducted about the aging process's complexity and the importance of vitamin D and vitamin B 12 [8-10]. In recent decades, studies have been conducted to determine the role of calorie restriction, chemicals, and intermittent fasting in cognitive function [9,10]. Other studies were conducted to ascertain the association between low body mass index (BMI) and malnutrition with dementia, Alzheimer's, and Parkinson's disease [11]. Poor dietary habits in the elderly have been linked to dementia, Alzheimer's disease, and Wernicke Korsakoff syndrome (WKS) [12,13]. Some of the essential nutrients, which include vitamin B complex, n-3 fatty acid, especially docosahexaenoic acid (DHA) from studies done, play a role in cognitive function [12,14-16]. In Parkinson's disease, which results from the degeneration of dopaminergic neurons, phytochemicals are believed to reduce the risk of neurodegenerative disease development over the years [17]. In multiple sclerosis, which is an inflammatory demyelinating disease of the central nervous system (CNS), riboflavin is essential in myelin formation, making riboflavin deficiency a risk factor for multiple sclerosis. This was based on the data from relevant clinical trials and experimental and case-control studies done from 1976 to 2017 [18]. WKS, which is characterized by neuronal loss, gliosis, and vascular damage in areas, surrounding the third and fourth ventricles and cerebral aqueduct, frequently occurs with thiamine (vitamin B1) deficiency in Western society. This syndrome is seen more frequently in alcoholic patients but can also occur from impaired nutrition from causes like gastrointestinal disease or acquired immunodeficiency syndrome (AIDS) [13].

In this article, we discussed how specific essential nutrients help in cognitive function and the prevention of certain neurocognitive diseases. We have done this study using the keywords “nutrition,” “brain degeneration,” and “neurodegeneration.” Data was collected from PubMed and Google Scholar database.

## Review

Vitamins and neurodegeneration

Most micronutrients (vitamins, minerals, essential amino acids, and essential fatty acids, including omega-3 polyunsaturated fatty acids [PUFAs]) have been studied in relation to cerebral functioning [19,20]. Thiamine (vitamin B1), which is essential for the maintenance of the brain function, when in decreased supply, results in different disorders of the nervous system (both peripheral and central nervous system defects). Though vitamin B1 deficiency is commonly found in alcoholics, it also occurs in non-alcoholics, making it challenging to diagnose [21]. It results in Wernicke’s encephalopathy, a neurological complication of thiamine deficiency, comprising of ophthalmoparesis with nystagmus, ataxia, and confusion. Early intervention with the provision of supplements with vitamin B1 is essential in preventing neurodegeneration caused by thiamine deficiency [13,22]. Vitamin C and E have been shown to act as nutritional antioxidants preventing the nervous system from free radical oxidative damage [23]. Vitamin E is known to help in the management of Alzheimer's disease, but more studies need to be conducted to know how it can be used to prevent the disease [23]. Some studies showed healthy older adults with low blood concentration of folate, vitamin C, riboflavin, and vitamin B12, performed poorly on tests of memory and nonverbal abstract thinking. There was significant improvement after supplementation with these vitamins [24]. Riboflavin, also known as vitamin B2, is a water-soluble vitamin that can be found in different foods and is essential for the nervous system's proper functioning. From randomized control trial and case-control studies, in which patients received riboflavin, it was found that riboflavin plays a crucial role in substrates production used for electron transport chain (ETC), which is essential in the supply of energy to the brain. Therefore, any defect in riboflavin would lead to neurodegeneration [18]. The list of food sources of vitamin B1, B2, and B12 are listed in Table 1 [25].

**Table 1:** Vitamins and Food Sources.

Vitamins	Food Sources
Vitamin B1	Fortified cereals, lean meats, dried beans, peas, and soy foods
Vitamin B2	Turkey, nuts, yogurt, eggs, milk, fortified cereals, and green leafy vegetables
Vitamin B12	Beef, poultry, fish, milk, eggs, cheese, salmon, sardines, and cereals

Vitamins have been shown to be essential in proper cognitive function. For people suffering from any neurodegenerative disease, they should be provided with food sources that contain these necessary essential vitamins to slow the progress of cognitive decline.

Role of dietary lipids in brain degeneration

**Figure 1:**
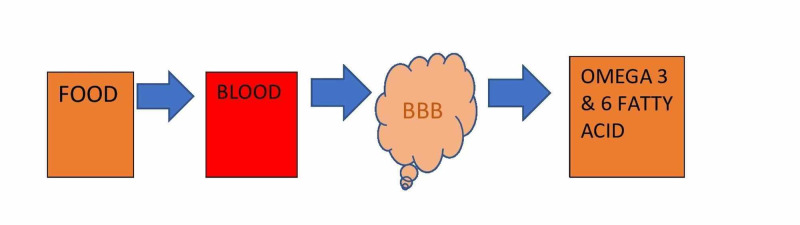
Illustration of fatty acid crossing BBB. BBB: Blood-brain barrier

Essential fatty acids (EFA) are crucial during infancy due to delay in brain development at this age group, and in the elderly due to the deterioration of brain functions. EFA, which is supplied through diet, must cross the blood-brain barrier (BBB), which is crucial in infancy and aging. When there is an imbalance in omega-3 and omega-6 PUFA, neurodevelopmental disorders occur by altering microglial activation that results in abnormal neuronal activity [26]. Figure 1 illustrates the fatty acid crossing BBB. Infants are born with immature BBB, and there are reports of structural changes in the BBB in Alzheimer's patients and aging [26]. However, more studies need to be conducted to determine how this structural change can lead to inadequate transport of fatty acids into the brain that leads to neurocognitive decline.

Reduced phospholipid levels from studies done have observed several neurodegenerative conditions. Phospholipids level was thought to play a role in spinocerebellar ataxia disorders. From a study conducted along with their fatty acid profiles, the concentration of phosphatidylethanolamine (PE), phosphatidylcholine (PC), and phosphatidylserine (PS) were measured in the brains of nine patients with predominantly inherited spinocerebellar atrophy type 1 (SCA-1) and eight patients with Friedreich ataxia (FA). It was found that PE, PS, and PC were decreased in the cerebellar and not occipital cortex in SCA-1 patients. However, there were decreased PS and PE levels in FA patients, but not PC in both the cerebellar and occipital cortices. The fatty acid composition of the brain phospholipids was altered both in FA and SCA-1 patients [27]. More studies need to be conducted to know exactly why this occurs, as the study is limited to how this resulted.

Some studies showed that the intake of high-fat diets like omega-6 and saturated fatty acids (SFA) results in poor cognitive function. At the same time, omega-3 fatty acids showed a protective effect against cognitive decline [28,29]. Greenwood and Winocour found that diets high in fat and lack of proper vitamins and minerals can worsen age-related cognitive decline when consumed later in life [28]. Exercise programs and diet interventions are necessary for the elderly to prevent cognitive decline [29]. From the Framingham heart study, it was suggested that 180mg or more dietary docosahexaenoic acid (DHA)/day, or approximately 2.7 fish servings per week, is associated with the risk of dementia [15].

DHA, which is a type of omega-3 fat, plays an essential role in cognitive function. A decreased level of DHA in the blood results in cognitive decline in the elderly [30]. Several studies have been conducted to correlate the effect of dietary DHA and cognitive function. In a study conducted, DHA increased cognitive function in a randomized controlled trial (RCT) that involved mentally healthy people older than 55. Daily DHA supplements for 24 weeks resulted in significantly lower paired associative learning errors than the placebo case. Regarding the effect of diet, nutritional support, vitamins, eicosapentaenoic acid (EPA), and DHA on memory cognition impairment, data showed low body mass index (BMI), and malnutrition correlated with mortality and dementia. At the same time, nutritional supports, and calorie-controlled diets play a protective role against Parkinson's disease, Alzheimer's, and cognitive decline [30]. Research lab utilized animal models and noticed that the balance of n-6 to n-3 fatty acid fed influences the composition of brain phospholipid fatty acid [4]. They concluded that biosynthetic control mechanisms regulating brain structural lipids might represent a normal physiological response of the brain, and this can be used for therapeutic for disorders that involve the degeneration of lipids of the brain [4,31]. Food sources of omega 3 and 6 are listed in Table 2 [32].

**Table 2:** Omega 3 and 6 Fatty Acid Sources.

Fatty Acid	Sources
Omega-3 fatty acid	Chia seeds, sardines, mackerel, anchovies, walnuts, salmon, and flax seeds
Omega-6 fatty acid	Corn oil, soybean oil, walnuts, cashew nuts, almonds, sunflower seeds, and mayonnaise

Lipids and Fatty acid role in the brain function from studies discussed indicate how essential the nutrients are for the maintenance of memory performance, cognitive function, and prevention of neurodegenerative diseases. These should be provided in the diets of aging individuals and those with neurocognitive disorders to reduce the progression of decline in cognitive function among these groups. 

Nutrition in dementia and multiple sclerosis

From current trends demographically, a significant rise in older people above 65 years of age in the world's population poses a significant risk of developing dementia [33]. Consumption of DHA food products has been shown from various studies to decrease neurodegeneration in dementia [15]. Several researchers analyzed the links between plasma DHA status and dementia. It was reported that patients with 30% less DHA in brain tissue had dementia due to Alzheimer’s disease. Feeding 30 subjects 640-800mg DHA for six months improved the performance level in 18 of 30 subjects (six of eight subjects without dementia and 12 of 22 subjects with dementia) [15]. Vascular dementia has been linked to various nutritional components, including lipids, folate, antioxidants, homocysteine, fish consumption, and vitamin B12. Vitamin E and C, fatty fish consumption was found to prevent vascular dementia. While elevated homocysteine, fried fish, and lower levels of vitamin B12 and folate caused an increased risk of vascular dementia [16].

Hyperhomocysteinemia, a metabolic disorder characterized by a systemic increase in homocysteine, occurs when B-group vitamins are deficient [34]. Folic acid, vitamin B6, and B12 deficiency have been associated with cognitive decline. From observational studies done, there is evidence of hyperhomocysteinemia strongly linked to impairment in old age. Even though elevated homocysteine levels lead to cognitive impairment, the underlying mechanism is unknown [34]. It was discovered from another study that malnutrition is associated with behavioral, psychiatric symptoms of dementia (BPSD). This study by Ai Kimura et al. showed nutritional status significantly associated with specific BPSD with a P-value of 0.016 [35]. Various studies were done between 1976 to 2017 to know the relation between multiple sclerosis (MS) and riboflavin. Findings collectively suggested that riboflavin is a useful option for treating MS patients and animal models [18].

Several studies have been conducted on the effectiveness of nutrition in dementia prevention, yet little is still known about its role in multiple sclerosis due to a limited number of studies done. Riboflavin has been said to help treat MS patients, but more studies need to be conducted to prove how, as it will be beneficial for patients suffering from this disease.

Nutrition involvement in Parkinson's disease

Parkinson's disease (PD) is a movement disorder with symptoms of rigidity, resting tremor, bradykinesia, and postural instability. Pathologically it is characterized by nigrostriatal dopaminergic neurons degeneration and the presence of Lewy bodies (abnormal aggregation of proteins) in the surviving neurons [36]. Flavonoids, a phytonutrient present in most vegetables and fruits, provide neuroprotective benefits in PD patients from research studies conducted [17,37]. The consumption of dairy products leads to a decrease in serum uric acids, and this decrease has been linked to Parkinson's disease and its duration [37]. However, more studies must be conducted to know how this occurs. From some research studies using 131,368 participants that were followed for 16 years, dietary components were analyzed using questionnaires. It was found that a diet high in vegetables, fruits, and fish was associated with reduced incidence of PD. In Parkinson's disease and Alzheimer's disease, the Mediterranean diet appears to be the preferred pattern of eating [38].

There are different classes of flavonoids, one of which baicalein, a flavone has been found to protect cells induced by E46K, which is a point mutation in α-synuclein responsible for Parkinson's disease. Baicalein also has neuroprotective benefits through antioxidant, anti-inflammatory, and antiapoptotic actions. In Table 3, different subclasses of flavonoids are listed that have positive effects on dopaminergic neurons [17]. From studies conducted, iron has also been linked to Parkinson's disease [39]. Alteration in the blood-brain barrier at the substantia nigra level increased uptake, and iron sequestering seen in PD. It was discovered that brain iron homeostasis is vital for the normal functioning of the brain [39].

**Table 3:** List of some examples of flavonoids subclasses with a positive effect in protecting dopaminergic neurons. EGCG: Epigallocatechin Gallate

Flavonoids Subclasses	Examples
Flavones	Baicalein, Luteolin
Flavonols	Quercetin, Rutin
Flavanones	Hesperidin, Naringin
Flavanols	EGCG
Isoflavones	Daidzein, Genistein
Anthocyanidins	Pelargonidin

Flavonoids have shown to be effective in preserving dopaminergic neurons, thereby preventing Parkinson's disease. However, long-term dietary intervention studies are essential to evaluate flavonoids' effectiveness in preventing this disease.

Nutrition and iodine deficiency in neurodegeneration

Iodine is a vital micronutrient incorporated into thyroid hormones that can be gotten through diet or iodine supplements. Iodine deficiency leads to various disorders throughout life [40]. Researchers formerly linked the abnormal mental and motor development caused by iodine deficiency to protein-energy malnutrition (PEM) in underdeveloped countries. Iodine deficiency is one of the world's most common preventable causes of cerebral palsy and mental retardation [6]. From experimental studies conducted, it was demonstrated that the cerebral cortex structure could be irreversibly disturbed in iodine deficiency, leading to abnormal migratory neuron patterns that are associated with cognitive impairment in children [40]. Iodine deficiency results in fetal and maternal hypothyroxinemia, which results in irreversible brain development impairment, occurs after 14 weeks gestational age, and could continue through the third trimester [6]. Iodization of salt, educational interventions with a strong emphasis on feeding nutrient-rich animal source food, and providing supplementations in populations with a diminished supply of the essential nutrients are essential in preventing neurodegeneration [41]. Emphasis on the adequate iodine nutrient supply for pregnant women is essential in preventing irreversible brain damage in children.

Limitations

This review's limitations include the following: only abstract was available for some studies, the inclusion of data more than 10 years, lack of a large sample size for some of the studies, and no quality assessment. We have excluded the papers published in other languages.

## Conclusions

Nutrition plays an essential role in preserving cognitive function and reducing neurodegenerative disease risk. We found out that vitamins, dietary lipids, and iodine all play an essential role in proper cognitive function. From studies conducted, sufficient supplies of these nutrients resulted in proper cognitive function. Diseases like Alzheimer's, Parkinson's, multiple sclerosis, and cerebral palsy were preventable with an adequate supply of these nutrients. Human and animal studies were done to know how an adequate supply of various nutrients in the diet helps reduce cognitive decline, especially in the elderly. These studies showed evidence in the prevention of some of these diseases from occurring. However, additional well-designed future studies need to be conducted to confirm how these nutrients work in preventing these diseases and what therapeutic supplements can be made available to prevent neurocognitive decline.
